# A GaN-HEMT Active Drain-Pumped Mixer for S-Band FMCW Radar Front-End Applications

**DOI:** 10.3390/s23094479

**Published:** 2023-05-04

**Authors:** Lorenzo Pagnini, Giovanni Collodi, Alessandro Cidronali

**Affiliations:** Department of Information Engineering, University of Florence, I-50139 Florence, Italy; lorenzo.pagnini@unifi.it (L.P.); alessandro.cidronali@unifi.it (A.C.)

**Keywords:** Volterra analysis, GaN devices, mixers, FMCW radar

## Abstract

This paper reports for the first time a drain-pumped (DP) mixer using Gallium Nitride (GaN) HEMT technology. Specifically, it describes a method aimed to predict the optimum bias conditions for active DP-mixers, leading to high conversion gain (CG) and linearity, along with the efficient use of the local oscillator drive level. A mixer prototype was designed and fabricated according to the discussed design principles; it exhibited a CG and an input third-order intercept point (IIP3) of +10dB and +11dBm, respectively, with a local oscillator power level of 20 dBm at about 3.7 GHz. In terms of gain and linearity, both figures exceed the documented limitations for the class of mixers considered in this work. To the authors’ best knowledge, this is the first DP mixer operating in the S-band. The prototype was also tested in a radar-like setup operating in the S-band frequency-modulated continuous-wave (FMCW) mode. Measurements carried out in the radar setup resulted in +39.7dB and +34.7dB of IF signal-to-noise-ratio (SNR) for the DP and the resistive mixers, respectively. For comparison purposes, a resistive mixer was designed and fabricated using the same GaN HEMT technology; a detailed comparison between the two topologies is discussed in the paper, thus further highlighting the capability of the DP-mixer for system applications.

## 1. Introduction

In many modern microwave receiving systems, a high level of dynamic range is a crucial feature, and a mixer is a functional block capable of influencing the overall system performance from this point of view. In the literature, it is well known that resistive mixers show the best performance in terms of linearity [1]. Nevertheless, their linearity features further benefit from using High Electron Mobility Transistors (HEMT) implemented in Gallium Nitride (GaN) technology as highlighted in the recent literature [2,3]; resistive mixers suffer from frequency conversion losses. For this reason, a low-noise amplifier (LNA) is introduced as first stage of the receiver chains, to compensate the losses and the subsequent added noise. However, since modern radar architectures make use of a large number of receivers and transmitter modules [4,5], the presence of the LNAs represents a significant increase in the overall system cost and complexity [6]. This is even more stringent when radar classification of UAV is of concern [7,8,9]. In addition, the realization of a low-complexity reader for transponder units [10,11] requires a highly dynamic receiver with a low count of subsystems. Therefore, the alleviation of their specifications is an open challenging topic.

In view of recent results [12], the problems previously described could be addressed by introducing GaN-based active mixer topologies that can provide linearity as well as gain. These considerations lead the authors to introduce an active DP mixer using GaN technology. In addition, the reduced complexity of such a mixer topology is considered as a fundamental feature, given the complexity in modern radar-system architectures.

The gate-pumped configuration is undoubtedly one of the most investigated among the active single-ended mixers. The latter has been proposed in a wide range of applications such as wireless communications [13,14], high-temperature applications [15], and radar systems [16].

With reference to the previous considerations and specifically referring to radar applications, the use of the DP topology is expected to provide some important benefits as explained in the following. The intrinsic RF-LO separation, a typical feature for the resistive configuration, is very relevant for radar applications given the adjacency between the RF and the LO signal frequencies. At the same time, the DP mixer topology preserves the CG, which is typical for the active configurations.

A demonstration of characterization and modeling of GaN-based devices in a mixer-like setup using the drain-driven HEMT was discussed in [17].

Concerning DP mixers specifically, very few were reported in the literature [18,19,20,21,22,23,24,25,26,27], of which only five are single-ended [18,19,20,21,24].

In [20], it was clearly shown that there is an improvement of both the conversion gain (CG) and input third-order intercept point (IIP3) with respect to the increasing level of LO power in CMOS-based technology; nevertheless, the LO power dynamic was limited to 10dBm.

Recently, an experimental-based analysis aimed at testing the impact of the GaN technology on the DP mixer was carried out [28]. In that work, the experimental data were firstly extracted from a packaged GaN-based device, and then elaborated to provide behavioral predictions about its operation as an ideal DP mixer. As expected, GaN technology allows to overcome the LO power level limitation of CMOS and other III–V semiconductor technologies, predicting an increase of both the mixer’s CG and IIP3. In the present work, the same device was considered for the design and implementation of a DP mixer. The mixer was characterized with single-tone and multi-tone measurements inside the S-band. To the authors’ best knowledge, this is the first DP mixer in this technology and the first DP mixer operating in the S-band. Among the DP mixers in single-ended topology, the present work reports both the highest CG and IIP3.

### 1.1. Optimum-Bias Methods for DP Mixers

In the literature, the problem of the optimum bias point in active mixers is well addressed for the gate-pumped topology, on which studies have also recently been carried out [29,30], but only a few works consider the DP topology. Concerning the gate-side, the general rule for achieving CG in DP mixers is to bias the device beyond its threshold voltage, and in [22], a method capable of predicting the bias value for achieving the maximum CG was proposed. Concerning the drain-side, the general rule for achieving CG is to set the drain-bias of the device close to the knee-voltage of its output characteristics, while to maximize it, the first Fourier coefficient of the time-varying waveform $g_{m}\left(t\right)$ has to be maximized. In [20], the coefficient was extracted and analyzed, but since it was a passive DP-mixer, the drain-bias optimization was not conceived. In addition, the extraction was performed starting from an approximate model. In this work, we propose a method that extracts the first Fourier coefficient of $g_{m}\left(t\right)$ directly from the physical device and for any desired drain-bias value, thus allowing for an experimentally based prediction of the optimum bias for active DP-mixers.

The analysis is inspired by the time-varying Volterra-series analysis [31,32,33,34,35]. This analysis models the effect of the LO signal, which is the source of strongly nonlinear mechanisms, as a time-modulation of the device’s intrinsic elements. As such, the time-domain waveforms of each component contain information regarding the global operation of the circuit. It is widely known that this approach is able to obtain very accurate predictions on field-effect transistors intermodulations, but at the cost of a considerable effort for the preliminary device parameter extraction [36].

In this work, rather than extracting the waveforms of all the circuit components, we extracted the ones of the components that provide the major contribution to the overall circuit operation. This made the analysis easier to be implemented, and at the same time, it provides the design stage with both qualitative and experimental insights. Recently, this approach resulted into a valuable tool for mixer behavioral predictions due to both its experimental basis and its simplicity [12,28,37,38]. The analysis of the present work is an extension of the approach used in [28] since in that case the Fourier coefficients were extracted for a single bias value. With respect to the harmonic-balance algorithm, this method has the following two main advantages. The first is its capability to get deep inside the mixer’s conversion mechanism, and the second is that it is based on experimental data, thus allowing to obtain pseudo-measurements already in the design stage of the mixer.

### 1.2. DP-Mixer in the Radar Context

Given the perspective of implementation in the context of radar systems, the mixer was then involved in a radar-like setup, and its operation was tested in the presence of an FMCW S-band signal. With the purpose of obtaining a term of comparison, a resistive mixer, which is often a part of the actual radar system front-end, was designed and fabricated using the same device. The mixers were compared while operating in the radar setup and in the same conditions of RF and LO signals. The adopted comparison criterion was the output IF signal-to-noise-ratio (SNR), of which the DP mixer showed the highest value, exceeding the passive counterpart by 3dB.

## 2. Analysis

It is well known in the literature that the most important contributions in general field-effect transistor applications come from the device drain-current [36,39]. In particular, in [20], it was shown that the device transconductance is the main contribution governing the CG in a DP mixer. In the following, the existing method for finding the optimum bias conditions for a passive DP mixer is considered, and then, the proposed extension for active DP mixers is discussed.

**Figure 1:**
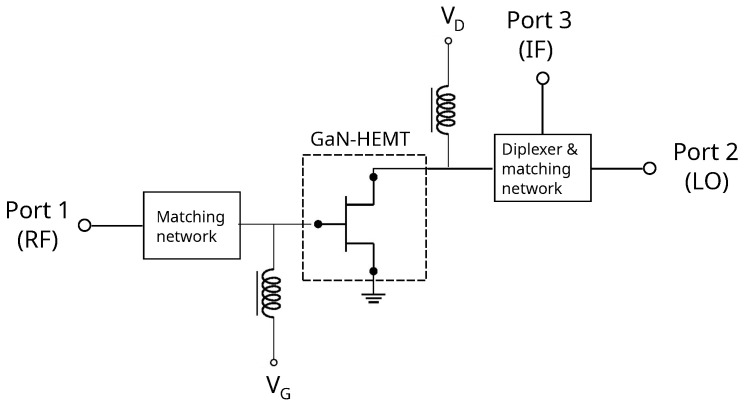
Schematic of the GaN-HEMT fundamental single-ended DP mixer.

The conceptual schematic of the DP mixer is reported in Figure 1. The data reported henceforth refer to the Wolfspeed’s CGH40006S GaN HEMT [40]. However, the frequency-independent feature of the adopted approach makes it also suitable for devices operating at higher frequencies.

### 2.1. Existing Method for Prediction of Optimum Bias Point in DP Mixers

The bias optimization methods for DP mixers reported in the literature only concern the passive configuration. Since the drain-bias is fixed, the optimization only concerns the device’s gate-side. As reported in the literature, the general rule for achieving CG is to bias beyond the device’s threshold voltage in such a way as to have an appreciable transconductance value. In particular, as found in [23], there exists a bias value that maximizes the CG, and it is found by considering the curve $g_{m}^{\prime }$ as a function of $V_{D}$, where

(1) $$g_{m}^{\prime }=\frac{\partial g_{m}}{\partial V_{D}}.$$

**Figure 2:**
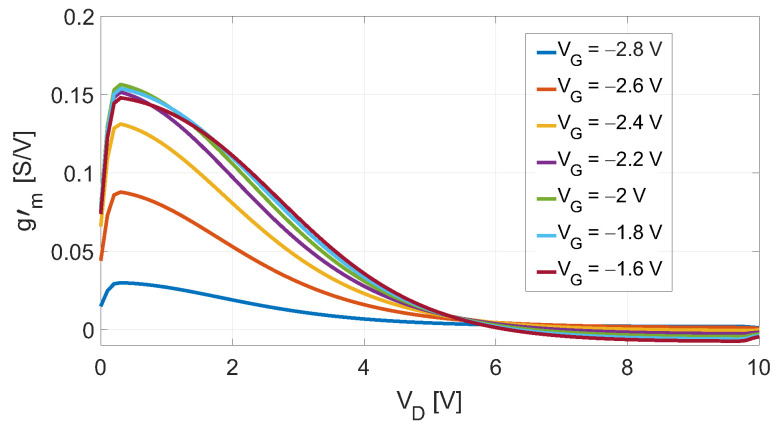
Simulated $g_{m}^{\prime }$ as a function of $V_{D}$ for different values of $V_{G}$.

After the extraction of $g_{m}^{\prime }\left(V_{D}\right)$ for various $V_{G}$ values as represented in Figure 2, the optimum $V_{G}$ for passive DP mixers is the one corresponding to the highest curve for $V_{D}\approx +0.2\;$V, which for the considered device is $V_{G}=-2\;$V.

### 2.2. Proposed Method for Prediction of Optimum Bias Point in DP Mixers

#### 2.2.1. Gate-Side

**Figure 3:**
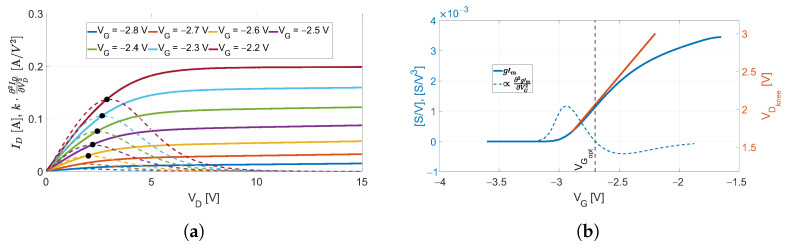
(**a**) Simulated $I_{D}$ and a term proportional to its second derivative $k\cdot \frac{\partial^{2}I_{D}}{\partial V_{D}^{2}}$ as functions of $V_{D}$ for different values of $V_{G}$. (**b**) Simulated $g_{m}^{\prime }$ and a term proportional to its second derivative $\alpha \cdot \frac{\partial^{2}g_{m}^{\prime }}{\partial V_{G}^{2}}$ as functions of $V_{G}$ for a fixed $V_{D}=+3\;$V; on the right-axis, the knee-voltage $V_{D_{knee}}$ is reported. The $V_{G_{opt}}$ resulted in ≈−2.65V.

Despite it was derived in the case of a passive DP mixer and its employment only concerns such devices [22,26,27], the validity of the method reported above can be extended to active DP mixers but with some adjustments. Firstly, the $g_{m}^{\prime }\left(V_{D}\right)$ is extracted for $V_{D}$ beyond the device’s knee voltage; for the device considered in this work, the knee voltage encompasses the region of $+1<V_{D}<+5\;$V. By looking at Figure 2, the existing method would consider the curve at $V_{G}=-1.6\;$V as the proper gate-bias since it is the highest in the $V_{D}$ range belonging to the knee region. However, such bias value would shift the knee voltage in the positive direction leading to a large LO dynamic for reaching the maximum CG. This is evident from Figure 3a, in which the device’s output characteristics are reported. The second derivative of each $I_{D}\left(V_{D}\right)$ curve, multiplied by a proper scale factor *k*, was also reported to help the visualization of the knee region. In fact, $\partial^{2}I_{D}/\partial V_{D}^{2}$ contributes only in this region, since both in the linear and in the saturation regions, the curves $I_{D}\left(V_{D}\right)$ are approximately lines. In this work, the $V_{D}$ values corresponding to the maxima of each derivative were associated to the device’s $V_{knee}$ values. The curves in Figure 2, as well as the ones in Figure 3a,b, resulted from the DC simulations of the schematic depicted in Figure 1; the device’s model was provided by the foundry.

A trade-off between CG and LO-power consumption is discussed in the following. For a better understanding of the concept, we consider a fixed value of $V_{D}$ belonging to the region of the device’s knee voltage, and we plot the $g_{m}^{\prime }\left(V_{G}\right)$ for such a value, together with the $V_{knee}\left(V_{G}\right)$, as shown in Figure 3b. The value $V_{G_{opt}}$ representing a good compromise for our purposes corresponds to the inflection point of the $g_{m}^{\prime }\left(V_{G}\right)$ function:

(2) $$\frac{\partial^{2}g_{m}^{\prime }}{\partial V_{G}^{2}}{|}_{V_{G}=V_{G_{opt}}}=\frac{\partial^{2}}{\partial V_{G}^{2}}\left(\frac{\partial g_{m}}{\partial V_{D}}\right){|}_{V_{G}=V_{G_{opt}}}=0.$$

In fact, by naming the required LO-voltage as $V_{LO_{req}}$, for every $V_{G}$ in the range $\left[\approx -2.9\;V,V_{G_{opt}}\right]$, the same $\Delta V_{G}$ would increase both the CG and the $V_{LO_{req}}$ by the same ΔCG and $\Delta V_{LO_{req}}$, while for $V_{G}>V_{G_{opt}}$, the same $\Delta V_{G}^{\prime }=\Delta V_{G}$ leads to $\Delta CG^{\prime }<\Delta CG$ and $\Delta V_{LO_{req}}^{\prime }=\Delta V_{LO_{req}}$. Therefore, by biasing the device with such a value, from one side, the CG is achieved because $V_{G}>V_{TH}$, and from the other side, the injected LO power is employed in the most efficient way. For the device under consideration, it results in $V_{G_{opt}}\approx -2.65\;$V. It is worth noting that the result of the present analysis is independent on the chosen $V_{D}$, since the shape of the $g_{m}^{\prime }\left(V_{G}\right)$ curves is approximately the same in the considered $V_{D}$ range.

#### 2.2.2. Drain-Side

**Figure 4:**
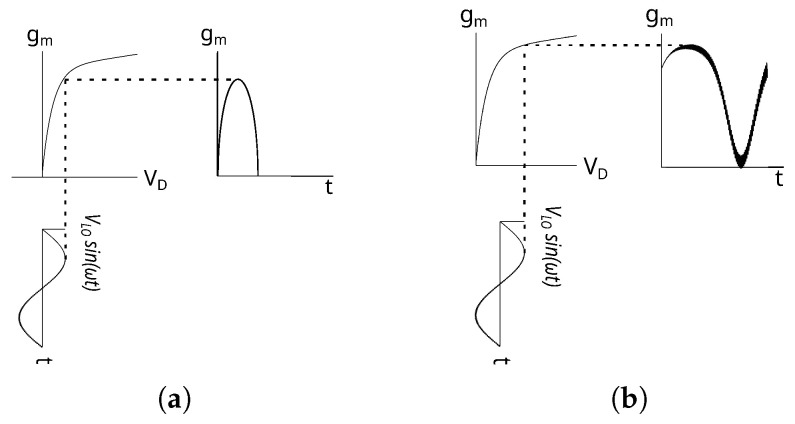
The GaN-HEMT device’s transconductance waveform $g_{m}\left(t\right)$ during the operation as (**a**) passive DP mixer and (**b**) active DP mixer.

Concerning the drain-side, the LO signal effect is a modulation of device’s transconductance, resulting in a time-varying waveform $g_{m}\left(t\right)$. As a consequence, the CG is directly proportional to its first Fourier coefficient $G_{m_{1}}$. In [20], a closed form of the Fourier coefficient was derived, but it is only valid for passive DP mixers. In fact, it was derived under the condition of zero drain-bias, resulting in a time-varying waveform $g_{m}\left(t\right)$, approximately a cosine function with 50% of the duty cycle, as shown in Figure 4a. Since the mixer of the present work is in the active configuration, a drain-bias greater than zero leads to a waveform significantly different with respect to the one resulting from a zero bias, as shown in Figure 4b.

In this work, we propose a method to maximize the first Fourier coefficient also for active DP mixers, as explained in the following. The results of the previous analysis led us to consider the $g_{m}\left(V_{D}\right)$ curve extracted for $V_{G}=-2.65\;$V. Therefore, the device’s $g_{m}\left(V_{D}\right)$ was experimentally extracted in conditions of gate-bias =−2.65V, as explained in the following. An RF tone of frequency 50MHz was injected at the gate-side while the power at the drain-side was measured for different $V_{D}$ values. The transconductance was then derived for each $V_{D}$ value from the ratio between the output current and the input voltage in the frequency domain, as described in [39]. Under the assumption of LO matching, the LO power at the port 2 of the circuit in Figure 1 is approximately the LO power at the device drain-side. Therefore, by finding the analytical function that best approximates the extracted $g_{m}\left(V_{D}\right)$ curve and by assuming a sinusoidal LO source, the $g_{m}\left(t\right)$ extraction is straightforward and the relative first Fourier coefficient is calculated analytically. It is worth noting that the same $g_{m}\left(V_{D}\right)$ curve is useful for the extraction of $g_{m}\left(t\right)$ for different drain-bias and LO power values. For the case of passive DP mixers, the same method can be applied by fixing the drain-bias at zero and considering only the variation of the LO dynamic.

**Figure 5:**
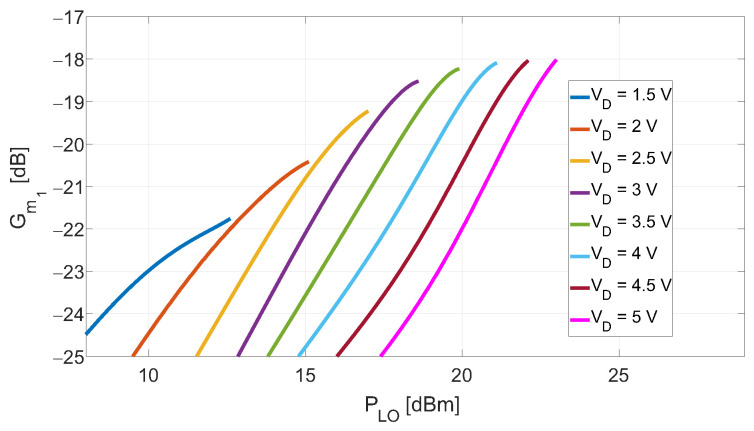
Experimentally extracted first Fourier coefficient $G_{m_{1}}$ of the waveform $g_{m}\left(t\right)$ as a function of $P_{LO}$ and $V_{D}$ for $V_{G}=-2.65\;$V. The predicted minimum LO power necessary to maximize the coefficient is ≈+20dBm, achievable when the device’s drain-bias is set at ≈+3.5V.

Figure 5 shows the results of the proposed method, in which the $V_{D}$ was varied between +1.5V and +5V and the $P_{LO}$ between +5dBm and +23dBm. For each considered $V_{D}$, the $P_{LO}$ was varied such as to keep the $V_{LO}$ in the positive region of $V_{D}$. This choice is due to the fact that $g_{m}\left(V_{D}\right)=0$ for $V_{D}<0$, and thus, this region is not expected to contribute to the CG. In addition, the region of $g_{m}\left(V_{D}\right)$ around $V_{D}=0$ is characterized by an abrupt change, and as such, the derivatives are expected to present oscillations, thus potentially increasing the intermodulations. The obtained results show the existence of a minimum LO power leading to the maximum $G_{m_{1}}$ when the drain-bias is set to ≈+3.5V. This bias point was adopted in this work since the main objective was to test the potential of the DP mixers using GaN technology. The maximum $G_{m_{1}}$ in the above conditions is obtained for $P_{LO}\approx +20\;$dBm.

Since the results presented in this subsection are based on an experimental-extraction procedure, a comment concerning the dispersion phenomena that affect GaN-based devices is necessary. The adopted $g_{m}$ extraction procedure was carried out dynamically both at the gate and at the drain-side, with the aim of partially reducing both the self-heating and the trap effects, which represent the main causes of dispersion phenomena. In fact, the dynamic of the RF signal injected at the gate-side was such as to substantially exceed the threshold voltage, and the drain voltage was set to zero in the transition between the values of $V_{D}\neq 0$, reproducing a “pulsed-like” scenario. In the light of the recent efforts in the characterization of these dispersion phenomena [41,42,43,44,45,46], the adoption of a purely pulse-based characterization technique would provide a more accurate isolation of the dispersion effects; consequently, the method introduced in this work could lead to the identification of a more accurate result. However, the adopted extraction procedure is considered suitable for the purposes of this work.

## 3. DP Mixer Design

Given the high LO power levels involved in the investigation of GaN DP mixers, as confirmed both by the previous work in [28] and by the analysis of Section 2, the GaN-HEMT CGH40006S was considered suitable for this work.

**Figure 6:**
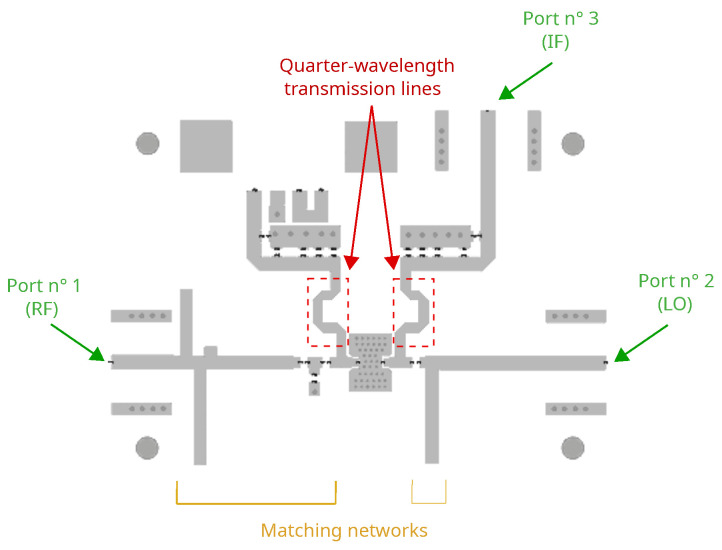
DP mixer layout in microstrip technology. The design was made for the operation in the S-band.

Figure 6 shows the topology of the mixer prototype. The DP developed in this work follows the general circuit topology depicted in Figure 1. It resembles somehow the development of the supply-modulated power amplifier [37], where the main difference consists in the device drain-bias, which was set to a value much larger than its knee voltage, and as expected, the envelope signal only weakly modulated the device transconductance. In the present work, the device drain-bias was set lower than the one in [37] and brought closer to its knee voltage, in such a way that the LO signal strongly modulates the device transconductance [18].

The RF and LO matching networks were designed to operate in the S-band. A quarter-wavelength transmission line was used as both gate-biasing circuit and IF short-circuit. Capacitors and open stubs were used, where the latter reproduced the inductors operation but with less losses. The two capacitors closest to the gate- and drain-side, respectively, play the role of both DC and IF-blocking, as well as contributing to the RF and LO matching. For the extraction of the output signal (port 3) at the intermediate-frequency (IF), a quarter-wavelength transmission line was designed. At the same port, the drain-bias was injected by means of an external bias-Tee.

## 4. DP Mixer Characterization

**Figure 7:**
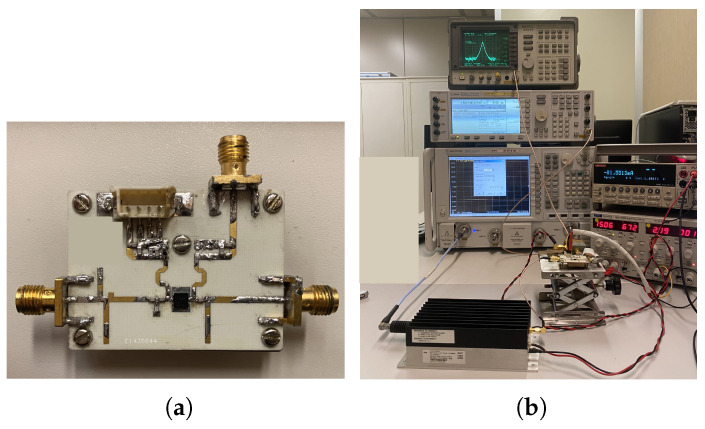
(**a**) Picture of the fabricated mixer prototype and (**b**) experimental setup for CG and IIP3 measurements.

Figure 7a shows the picture of the fabricated circuit, which was implemented in a 20 mil RO4350 laminate. The measurement procedure of the mixer was performed as follows. A single-tone characterization was carried out, during which the CG was measured as a function of the LO power and frequency for different values of gate-bias, followed by a two-tone characterization from which the IIP3 value was extrapolated. The experimental setup is represented in Figure 7b. The LO signal was generated by a synthesized source with the external amplifier required to get enough LO power, while the RF signal was generated by an arbitrary signal generator. The output power was measured by a spectrum analyzer. Finally, the mixer was involved in a radar setup to demonstrate its operation in an actual context.

### 4.1. Single-Tone Characterization

**Figure 8:**
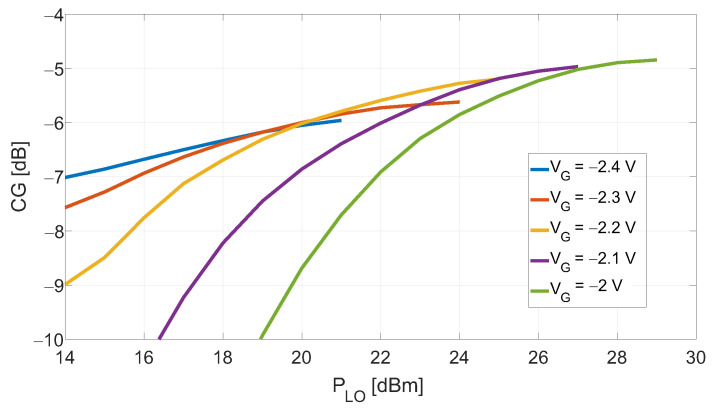
DP mixer’s measured CG as a function of $P_{LO}$ with the gate-bias as parameter and drain-bias +0.2V.

The DP mixer prototype described in Section 3 was first characterized with a drain-bias close to zero with the purpose of proving the limitations of the existing gate-bias method, as stated in Section 2. The passive DP mixer was chosen for this test since, being $V_{D}$ close to zero, the device’s transconductance only weakly depends on $V_{G}$, thus keeping the reflection coefficient at the gate-side approximately constant in the considered $V_{G}$ range. This allows us to perform a consistent comparison between the $CG(P_{LO})$ curves obtained for different $V_{G}$ values as reported in Figure 8. As it emerges from the results, the optimum gate-bias value according with the existing method allows to achieve the maximum CG, but at the cost of a large $P_{LO}$ of ≈+29dBm for the device employed in this work. In particular, it can be noticed that increasing $P_{LO}$ from +21dBm to +29dBm, the CG increases just over 1dB.

**Figure 9:**
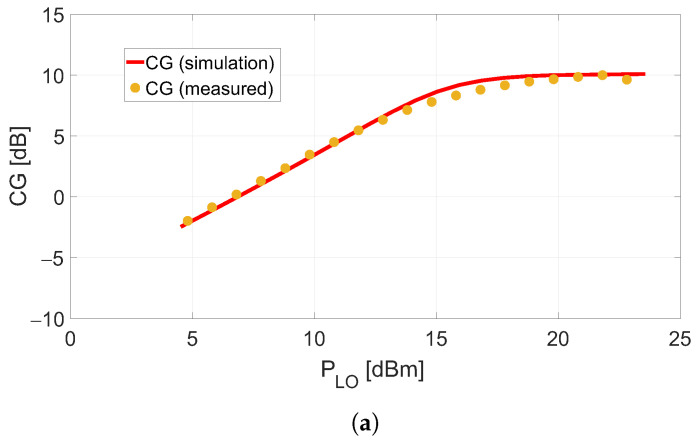

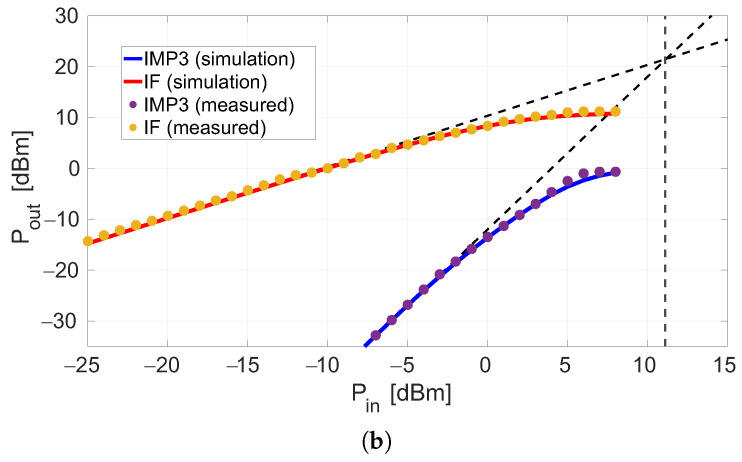
DP mixer’s single-tone and two-tone characterization in the gate- and drain-bias conditions of −2.65V and +3.5V, respectively. (**a**): Simulated and measured CG as a function of $P_{LO}$. (**b**): Simulated and measured output power of IF and IMP3 tones as a function of the input power for $P_{LO}=+20\;$dBm.

The results of the single-tone characterization for the active DP mixer in the bias conditions proposed in this work are shown in Figure 9a. The DP mixer reached CG=+10dB for the $P_{LO}$ value predicted by the analysis, about +20 dBm, with RF and LO signals at 3.7 and 3.74 GHz, respectively. The same figure also reports the Harmonic-Balance (HB) simulation results, which involve the layout characterization made by an electromagnetic simulator.

### 4.2. Two-Tone Characterization

Under the above conditions of bias and $P_{LO}$, a two-tone signal of frequencies 3.709±0.002GHz was injected at the port 1, and its power was swept from −25 to +8dBm. Figure 9b reports the output power at the IF frequency of 43MHz and the third-order intermodulation product (IMP3) power at 45MHz as functions of the input power. From the curves, an IIP3 value of about +11dBm was extrapolated. The same figure also reports the HB simulation results.

Both the CG and the IIP3 results exceed the values reported so far in the literature for single-ended DP mixers.

### 4.3. FMCW Radar-Mode Setup

**Figure 10:**
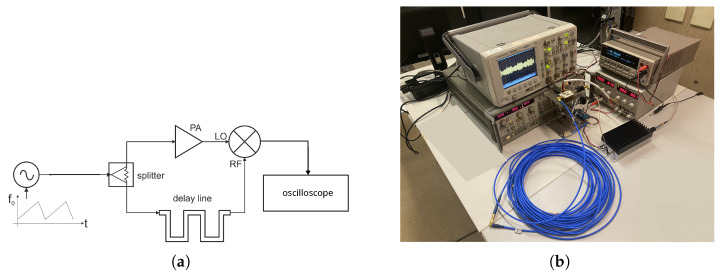

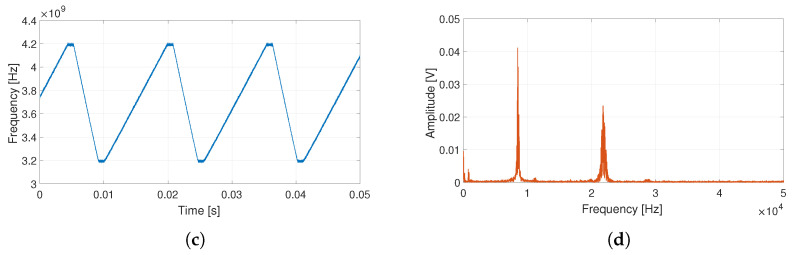
(**a**) Conceptual schematic of the radar setup mode; (**b**) experimental radar setup mode; (**c**) generated FMCW signal; (**d**) output spectrum of the DP mixer.

An FMCW radar-mode setup test bench was implemented with the aim to demonstrate the DP mixer operation in an emulated radar context. Figure 10a shows the conceptual schematic, and the picture in Figure 10b shows the adopted experimental setup. A frequency-modulated signal around 3.7 GHz, represented in Figure 10c, was generated by the sweep oscillator and injected in a splitter. One of the two output ports of the splitter was connected to the external amplifier, thus emulating the LO signal, and the other one was connected to the delay line, thus emulating the return RF signal.

The adopted setup allowed us to obtain an output IF signal of a few kHz as explained in the following. The delay line introduced a time delay of 85ns, and since the swept oscillator constraints concerning the minimum raise and fall times of the generated ramp were 10ms and 4ms, respectively, a frequency-modulated signal of 1 GHz bandwidth was used. In this way, the IF signals of frequencies about 8.5 kHz and 21.25 kHz were predicted.

Figure 10d shows the resulting output spectrum, confirming the predictions discussed above. It is worth noticing that the signal at 21.25kHz results in more dispersion since it is related to the falling side of the ramp, which is not perfectly controlled by the instrument.

## 5. DP and Resistive Mixer Comparison

With the purpose of providing a term of comparison in the radar context, a resistive mixer was designed and fabricated using the same technology. The DP and the resistive mixers share the same circuit topology, only differing in the fact that the first operates with the RF signal injected at the port 1 and the LO signal at the port 2, and the latter operates with these two signals exchanged with each other [21,24]. This allowed us to easily perform a consistent comparison since the same circuit layout of Figure 6 can be adopted for the design of the resistive mixer. Since the drain-bias is zero for a resistive mixer, a proper capacitor of the drain-side’s matching network was selected in order to optimize the RF matching.

### 5.1. Resistive Mixer Characterization

**Figure 11:**
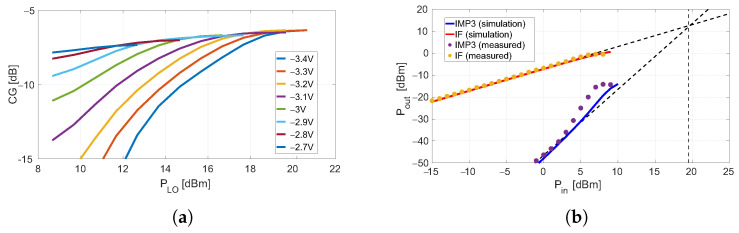
(**a**) Measured CG as a function of the LO power with the gate-bias as parameter for the resistive configuration; (**b**) simulated and measured output power IF and IMP3 tones as a function of the input power for the resistive configuration, at gate-bias of −3 V, and LO = +16 dBm.

The CG of the resistive mixer was measured as a function of the LO power and for different bias values to search for a good compromise between CG and $P_{LO}$ consumption. The results are reported in Figure 11a, and the selected combination is +16dBm and −3V for the $P_{LO}$ and $V_{G}$, respectively. Under these conditions, a two-tone signal at the same frequencies as for the DP-configuration was applied at the port 2. The input power was swept from −15 to +10dBm, and Figure 11b shows the output IF and IMP3 power tones as functions of the input power. The measured value of IIP3 was about +19.5dBm. As for the DP-mixer, the measurement results were supported by the HB simulations. The simulation results are not shown in Figure 11a for clarity.

### 5.2. Comparison for Radar-Mode Operation

**Figure 12:**
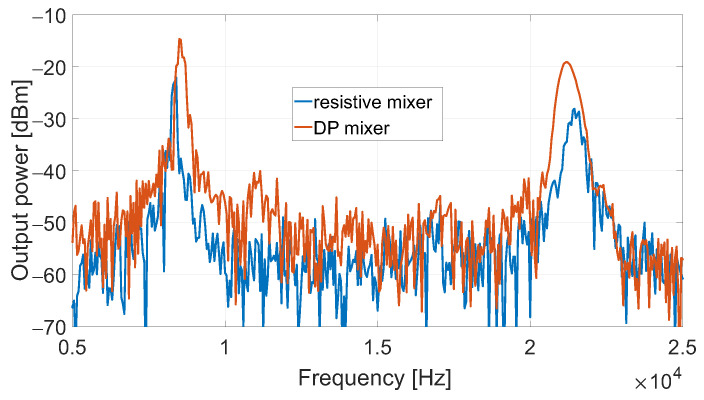
Output spectrum of the DP and resistive mixers while operating in radar setup.

The resistive mixer was involved in the same radar setup as in Figure 10b and the same ramp as in Figure 10c was employed. The output spectrum of the resistive mixer is reported in Figure 12 overlapped with the one of the DP mixer. The measurements were carried out while the two mixers operated with the same RF and LO power levels, corresponding to about −18 and +10dBm, respectively. The above conditions allowed us to carry out a clean comparison between the two mixers, but they clearly penalized the DP mixer. Nevertheless, it showed an SNR of +39.7dB versus the +34.7dB of the resistive mixer, demonstrating that it is a valid alternative even in conditions of lower LO power than its optimal value.

Differently from Section 4, in which the limitations of the mixer’s performance had to be tested and thus its was driven with high LO power levels, in the present section, the proper operation of the mixer was also demonstrated in more relaxed conditions of LO. The results could be significant in view of its implementation for applications with stringent power requirements.

## 6. Conclusions

In this work, the first DP mixer in GaN is presented, showing +10dB of CG and +11dBm of IIP3. The high drain-to-source breakdown voltage of this technology is at the basis of both high gain and high linearity. In Table 1, the state-of-the-art performance of single-ended DP mixers in terms of gain and linearity is reported, among which this work shows by far the best performance. A figure-of-merit aimed to quantify the efficiency with which the $P_{LO}$ is employed is reported in the same table. The mixer was also demonstrated in actual radar setup mode. In both the multi-tone and radar modes, the DP mixer was compared with the resistive mixer. In the latter mode, the DP exhibited a better SNR, respectively, 39.7dB versus 35.7dB. This component results in a good candidate for radar systems with many channels where complexity and performance are of concern.

## Figures and Tables

**Table 1:** State-of-the-art of single-ended drain-pumped mixers.

Work	Technology	Frequency-Band	CG [dB]	IIP3 [dBm]	V${}_{D}$ [V]	P${}_{d}$ [mW]	P${}_{LO}$ [dBm]	P${}_{LO}$-CG [dBm]
[18]	GaAs MESFET	X	+1.5	-	+0.7	17 *	+10.5	+9
[19]	GaAs pHEMT	Ku	+2.5	0	+1.1	-	+15	+12.5
[20]	SOI CMOS	Ka	−4.6	+4 *	0	0	+7.5	+11.6
[21]	GaAs mHEMT	G	−7 *	-	+0.2	0.55	+5	+12
[24]	InP HEMT	F	−6.5	-	0	0	+6	+12.5
This work	GaN HEMT	S	+10	+11	+3.5	87	+20	+10

* Estimated from plots.

## Data Availability

No additional data are available.
